# The impact of acute bouts of interval and continuous walking on energy‐intake and appetite regulation in subjects with type 2 diabetes

**DOI:** 10.14814/phy2.13524

**Published:** 2017-12-06

**Authors:** Ida A. Müller, Anne‐Sophie Wedell‐Neergaard, Thomas P. J. Solomon, Kristian Karstoft

**Affiliations:** ^1^ The Centre of Inflammation and Metabolism and the Centre for Physical Activity Research Rigshospitalet University of Copenhagen Copenhagen Denmark; ^2^ School of Sport, Exercise, and Rehabilitation Sciences University of Birmingham Birmingham United Kingdom; ^3^ Institute of Metabolism and Systems Research (IMSR) University of Birmingham Birmingham United Kingdom; ^4^ Department of Clinical Pharmacology Bispebjerg Hospital University of Copenhagen Copenhagen Denmark

**Keywords:** Appetite, exercise, gastric emptying, gut hormones, type 2 diabetes

## Abstract

In healthy subjects, it has been suggested that exercise may acutely suppress energy‐intake and appetite, with peak intensity being an important determinant for this effect. In subjects with type 2 diabetes (T2D), the effect of exercise on appetite‐related variables is, however, virtually unknown. We aimed to assess the effects of two exercise interventions, differing with regards to peak intensity, on energy‐intake, satiety and appetite‐related hormones in subjects with T2D. Thirteen subjects with T2D completed three 60‐min interventions with continuous measurement of oxygen consumption in a randomized and counterbalanced order: (1) Control, (2) Continuous walking (CW; intended 73% of VO_2_peak), (3) Interval‐walking (IW; repeated cycles of 3 min slow [54% of VO_2_peak] and 3 min fast walking [89% of VO_2_peak]). Forty‐five minutes after completion of the intervention, a 3‐h liquid mixed meal tolerance test (MMTT, 450 kcal) with regular satiety assessments and blood samples for appetite‐related hormones commenced. An ad libitum meal was served after the MMTT, with subsequent calculation of energy‐intake. Moreover, free‐living diet records were completed for the following ~32 h. Exercise interventions were well‐matched for mean oxygen consumption (CW = 77 ± 2% of VO_2_peak; IW = 76 ± 1% of VO_2_peak, *P* > 0.05). No differences in appetite‐related hormones or energy‐intake were found (*P* > 0.05 for all comparisons). IW increased fullness compared to Control shortly after the intervention (*P* < 0.05) and tended to reduce hunger 2 h into the MMTT compared to CW and Control (*P* < 0.10). In conclusion, a single exercise session does not affect energy‐intake during the following ~4–36 h in subjects with T2D. However, satiety may be affected up to ~3 h after the exercise session, dependent on peak intensity.

## Introduction

Lifestyle changes, including weight loss, are a first line treatment for subjects with type 2 diabetes (T2D) (American Diabetes Association, 2014). Weight loss is recommended for all overweight/obese T2D subjects since it improves insulin sensitivity, glycemic control, and other cardiovascular risk factors (Ross et al. 2000; Coker et al. 2009). Moreover, post hoc analyses from the LookAHEAD study recently showed that a large weight loss, primarily based on lifestyle changes, may reduce the risk of macrovascular complications in subjects with T2D (Look AHEAD Research Group, 2016).

Weight loss may be induced by increased physical activity (Shaw et al. 2006). This may theoretically be dependent both on the extra calories burned during and after exercise (Karstoft et al. 2016) and on the acute effects of exercise on energy intake (Martins et al. 2008). In relation to the latter, it has been suggested that exercise bouts may acutely reduce appetite in both lean (King et al. 1994) and obese (Ueda et al. 2009b) healthy subjects. These findings have largely been ascribed to exercise‐induced changes in appetite‐related hormones with acute exercise‐induced increases in appetite‐suppressing hormones like peptide YY (PYY), pancreatic polypeptide (PP), and incretins and decreases in appetite‐increasing hormones like ghrelin (Schubert et al. 2014).

Although we (Knudsen et al. 2013, 2014) and others (Erdmann et al. 2005) have suggested that subjects with T2D and hyperglycemia have altered appetite regulation compared to healthy subjects, only two studies have, to our knowledge, examined the effects of acute exercise on appetite regulation in T2D subjects (Knudsen et al. 2013; Heden et al. 2016). These studies have both evaluated satiety and appetite‐related hormones, but studies examining energy‐intake after exercise in subjects with T2D are lacking. This is important to assess since discrepancies between satiety and appetite‐related hormones on one side and energy‐intake on the other have previously been reported (Bilski et al. 2013; King et al. 2013; Holliday and Blannin 2017).

The intensity of exercise has been acknowledged as an important determinant for the appetite‐regulating effects of exercise (Hazell et al. 2016). As such, Ueda et al. (2009a) found that aerobic exercise with higher intensity increases PYY more than aerobic exercise with lower intensity in lean, healthy males and Sim et al. (2014) have shown that high‐intensity intermittent exercise with anaerobic peak intensity reduces ad libitum meal intake shortly after the exercise session compared to continuous exercise with moderate intensity and no exercise in overweight, sedentary males. Moreover, free‐living diet records indicated that the suppressive effect of high‐intensity exercise on energy intake was sustained for 2 days (Sim et al. 2014). Despite free‐living dietary records are prone to inaccuracies and underreporting (Hill and Davies 2001; Samuel‐Hodge et al. 2004), these findings are interesting, since it has been documented that training interventions with higher intensity results in larger decrease in body weight compared with training interventions with lower intensity although training‐derived energy expenditure is comparable (Shaw et al. 2006). In this respect, we have shown that 4 months of interval walking (IW) training induces weight loss, whereas energy expenditure matched continuous walking (CW) training does not in subjects with T2D (Karstoft et al. 2013), and hypothesis‐generating data have suggested that IW reduces free‐living energy intake the day after exercise compared with CW in subjects with T2D (Karstoft et al. 2014). Altogether, these data indicate that peak exercise intensity is important for the magnitude of training‐induced weight loss, and suggests that differential appetite regulation and therefore energy‐intake following single exercise sessions with different peak exercise intensity may be an important contributor to the differential improvements in body composition. Since this, however, has never been tested in subjects with T2D, the objective of this study was to assess the acute effects of exercise with lower (CW) and higher (IW) peak intensity on energy‐intake, satiety and appetite‐related hormones in subjects with T2D. We hypothesized that IW, in opposition to CW, would decrease energy‐intake and increase satiety compared with the control situation (CON), and that this would be dependent on differential effects on appetite‐related hormones.

## Materials and Methods

### Subjects

Subjects with T2D (American Diabetes Association, 2016) were recruited to the study. Inclusion criteria were age above 30 years and a BMI between 25 and 40 kg/m^2^. Exclusion criteria were pregnancy, smoking, contraindication to increased levels of physical activity (Pedersen and Saltin 2015), eating disorders, treatment with exogenous insulin, and evidence of any diabetic complication. Subjects who were potentially eligible underwent a screening consisting of a medical history and examination, an oral glucose tolerance test (OGTT), a Dual X‐ray Absorptiometry (DXA) scan (Lunar Prodigy Advance; GE 253 Healthcare, Madison, WI) and a walking VO_2_peak test on flat ground with portable indirect calorimetry (Cosmed, K4B2, Rome, Italy) as previously described (Karstoft et al. 2014). Following familiarization to the exercise interventions (see below) and a VO_2_max test (walking with incremental inclination) with stationary indirect calorimetry (Cosmed Quark, Rome, Italy) on a treadmill (Katana Sport, Lode, Groningen, the Netherlands) as previously described (Karstoft et al. 2013), subjects completed a leisure time physical activity questionnaire (Taylor et al. 1978).

The familiarization to the exercise interventions was performed on the treadmill (Katana Sport) with indirect calorimetry (Cosmed Quark) and was based on the VO_2_peak obtained. The CW intervention was intended to be performed with 73% of VO_2_peak and the individually appropriate speed was found by stepwise increasing the treadmill speed until the intended oxygen consumption rate was reached. Likewise, the IW intervention was intended to be performed with 54% and 89% of VO_2_peak during slow and fast walking, respectively, and the individual speeds were found by stepwise increasing the treadmill speed.

Informed written and oral consent was acquired from all the subjects prior to any procedures and the study was approved by the regional ethical committee (H‐15008542) and registered at www.ClinicalTrials.gov (NCT02592616).

The sample size was based on the ad libitum energy intake in the study by Sim et al. (2014). In this study, ad libitum energy intake was 3199 ± 1642 kJ (mean ± SD) following the control intervention and 2602 ± 1086 kJ following the high‐intensity, intermittent exercise intervention. The correlation coefficient (Pearson) between the ad libitum energy intake in the control versus the high‐intensity, intermittent exercise intervention was 0.91 (information obtained by the corresponding author). As such, with *α* set at 0.05 and a selected power (1 − *β*) of 0.80, analysis (G*Power, v3.1.9.2, Düsseldorf, Germany) indicated that 13 subjects should complete this study (resulting in an actual power of 0.82).

### Trials

Subjects were included in a cross‐over study with three trials, separated by 1 week and differing only with regards to the following 60‐min interventions: a control intervention (CON), a CW intervention and an IW intervention. Trials were performed in a randomized and counterbalanced order (www.randomization.com: Seed 17043), without blinding of subjects or investigators. Each trial was 2 days long, with the intervention performed on the first day.

Subjects were instructed to avoid vigorous exercise and pause antidiabetic medication and paracetamol from 48 h before and to the end of each trial. In the 24 h preceding the first intervention day, subjects started diet records with the instruction to eat as normal as possible and to refrain from alcohol and caffeine containing liquids. At subsequent trials, subjects were handed a copy of the diet record and were instructed to follow this for the 24 h preceding each intervention day.

### Intervention day

At the intervention day, subjects consumed a standardized solid breakfast consisting of a wheat bun (100 g) with jam (20 g) with a caloric content of 1243 kJ (69 E% carbohydrate, 13 E% fat, 18 E% protein). The breakfast was ingested with ad libitum water between 90 and 120 min before arrival at the laboratory. Upon arrival (at 9:00 am), subjects were weighed, an antecubital vein catheter was placed and the 60‐min intervention began. CW/IW was performed at a treadmill (Katana Sport), whereas subjects were sitting on a chair during the entire duration of CON. During all the interventions, continuous measurement of oxygen consumption (Cosmed Quark) and heart rates (Cosmed wireless heart rate monitor) was performed. Moreover, subjects rated the perceived exertion (RPE) (Borg 1982) at halfway and in the end of the exercise bout (in IW both during fast and slow intervals).

Upon completion of the intervention, subjects rested for 45 min after which a 3 h liquid mixed meal tolerance test (MMTT: Nestlé Resource Komplett Näring 1.5, Frankfurt, Germany, 300 mL, 450 kcal [55E% carbohydrates, 15E% protein and 30E% fat]) commenced. Subjects were seated throughout the duration of the MMTT. The MMTT was spiked with 1.5 g paracetamol in order to allow for assessment of gastric emptying (Medhus et al. 1999). After the MMTT, the antecubital catheter was removed and subjects were taken to a quiet room and served an ad‐libitum meal consisting of either meat sauce with mashed potatoes or meat balls in curry with rice (each subject had to choose the same dish in all trials) and with ad libitum water. Meal sizes were larger than subjects could consume and subjects were instructed to eat until they felt comfortably full. The same investigator gave this instruction in all trials. Plates were weighed before and after the ad libitum meal and the consumed meal energy content was calculated. After completion of the ad libitum meal, subjects left the laboratory but completed free‐living diet records until the end of the following day.

A four point (assessing hunger, prospective food consumption, nausea, and fullness) validated satiety questionnaire (Flint et al. 2000) was completed by the subjects regularly during the intervention day (before starting the intervention, before the MMTT and then hourly during the MMTT) and during the following day (immediately before and after breakfast, lunch, and dinner).

### Blood sampling and analyses

Blood samples were taken before and during (halfway through and in the end of) the intervention, and every 15th minute until the ad libitum meal was served. Lactate was measured in whole‐blood at a bedside analyzer (ABL 7 series, Radiometer, Herlev, Denmark) and all other blood samples were kept on ice until centrifugation (15 min, 2000 g, 4°C), after which plasma was stored at −80°C until analyses.

Samples for active (acylated) ghrelin were collected in EDTA‐coated tubes preserved with 4‐(2‐aminoethyl) benzenesulfonyl fluoride hydrochloride (Sigma, Copenhagen, Denmark). Plasma for ghrelin analysis was acidified to prevent hydrolysis according to guidelines (Hosoda and Kangawa 2012). Active ghrelin was analyzed using radioimmunoassay (RIA) kits (Millipore, St. Charles, MO).

Samples for leptin, PP and PYY were collected in EDTA‐coated tubes and analyzed using a commercial Luminex^®^ kit (Milliplex, Human gut hormone panel, Millipore).

Samples for paracetamol were collected in lithium‐heparin‐coated tubes and analyzed using an enzyme multiplied immunoassay technique (Cobas 8000, Roche Diagnostics, IN).

### Calculations

Mean oxygen consumption and heart rates were calculated during the entire intervention. Moreover, mean of the last minute of slow/fast intervals were calculated during IW.

Energy expenditure during the interventions was calculated assuming a uniform oxygen equivalent of 20.2 kJ/L O_2_ (McArdle et al. 2001).

Energy intake from diet records was calculated using an online diet registration tool (www.madital.dk), as previously described (Karstoft et al. 2014).

### Statistics

Variables measured during the exercise interventions (walking speed, rate of perceived exertion and oxygen consumption + heart rates) were compared using Student's paired *t*‐test. Other variables were compared using one‐way (1W), repeated‐measures (RM) analyses of variance (ANOVA) with Bonferroni‐corrected post hoc tests. Data are presented as mean ± SEM and analyzed using Prism v6 (Graphpad, San Diego, CA), with *P* < 0.05 considered significant.

## Results

In total, 14 subjects were included in the study. One subject did not finish the study due to personal reasons, leaving 13 subjects completing all trials. Baseline characteristics for these 13 subjects are shown in Table 1.

**Table 1:** Baseline characteristics

*N*	13
Sex (M/F)	8/5
Age (year)	65 ± 2
Time since diagnosis (year)	9 ± 2
MLTPAQ (kcal/day)	287 ± 54
Glucose‐lowering medication (*n*)	
No medication	2
Metformin	11
Sulfonylureas	3
GLP‐1 analogs/DPP‐4 inhibitors	4
Body composition	
Body mass (kg)	102 ± 5
BMI (kg/m^2^)	33 ± 1
Lean body mass (kg)	66 ± 3
Fat mass (kg)	37 ± 3
Fitness variables	
VO_2_max (mL O_2_/min per kg)	25.3 ± 1.1
VO_2_max (L O_2_/min)	2.6 ± 0.1
VO_2_peak (mL O_2_/min)	2.1 ± 0.1
VO_2_peak (% of VO_2_max)	81 ± 3
Glycemic control	
Fasting glucose (mmol/L)	7.3 ± 0.3
Fasting insulin (pmol/L)	116 ± 14
Two‐hour OGTT glucose (mmol/L)	11.5 ± 1.0
HbA1c (mmol/mol)	48 ± 2

Data are mean ± SEM.

MLTPAQ, Minnesota Leisure Time Physical Activity Questionnaire (Taylor et al. 1978); GLP‐1, Glucagon‐like Peptide‐1; DPP‐4, Dipeptidyl peptidase‐4; VO_2_max, maximal oxygen consumption rate; VO_2_peak, peak oxygen consumption rate; BMI, Body Mass Index; OGTT, oral glucose tolerance test; HbA1c, Hemoglobin A1c.

No differences between trials were seen in energy intake prior to the intervention day, and body weight did not differ between trials (data not shown).

### Intervention variables (Table 2)

**Table 2:** Exercise characteristics

Oxygen consumption rate (mL/min)	CW	IW
Mean	1603 ± 117	1577 ± 110
Slow walking intervals		1197 ± 81^#^
Fast walking intervals		1919 ± 137^#^
Heart rate (bpm)		
Mean	106 ± 4	110 ± 5
Slow walking intervals		100 ± 4^#^
Fast walking intervals		120 ± 5^#^
Walking speed (km/h)		
Mean	4.8 ± 0.2	4.4 ± 0.2*
Slow walking intervals		3.1 ± 0.2^#^
Fast walking intervals		5.6 ± 0.3^#^
Rate of perceived exertion (a.u.)		
Mean	11.7 ± 0.5	11.8 ± 0.4
Slow walking intervals		10.8 ± 0.4^#^
Fast walking intervals		12.8 ± 0.5^#^

Data are mean ± SEM.

Bpm, Beats per Minute; CW, Continuous Walking; IW, Interval Walking. Slow/fast walking intervals includes data from the last min of each interval. Variables were compared using Student's paired *t*‐tests. Statistical differences (*P* < 0.05) are indicated by * (CW vs. IW), # (IW slow/fast intervals vs. CW).

Mean oxygen consumption rates did not differ between CW (77 ± 2% of VO_2_peak) and IW (76 ± 1% of VO_2_peak, *P* > 0.05), whereas mean oxygen consumption rate was higher during CW compared to slow IW intervals (58 ± 1% of VO_2_peak, *P* < 0.05) and lower during CW compared to fast IW intervals (92 ± 2% of VO_2_peak, *P* < 0.05).

No differences between CW and IW were seen for mean heart rates or RPE, whereas mean walking speed was higher during CW compared with IW (*P* < 0.05). During slow IW intervals, heart rates, RPE, and walking speed were lower compared to mean CW, whereas, during fast IW intervals, heart rates, RPE, and walking speed were higher compared to mean CW (*P* < 0.05 for all comparisons).

Lactate levels were higher during the IW intervention (1.9 ± 0.4 mmol/L) compared to both CW and CON (1.1 ± 0.1 mmol/L and 1.0 ± 0.1 mmol/L, respectively; *P* < 0.05 for both), whereas no differences were seen between CW and CON (*P* > 0.05).

### Satiety variables (Fig. 1)

No baseline differences were seen in satiety variables between trials.

**Figure 1:**
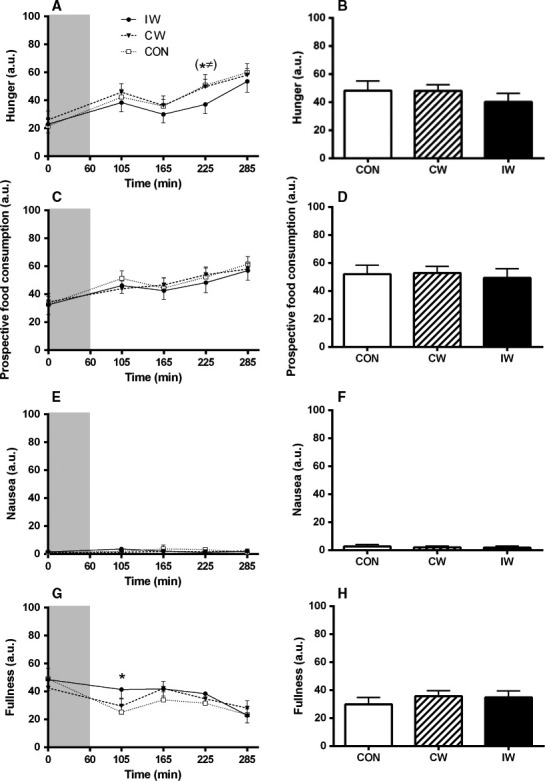
Satiety variables assessed using visual analog scales before and after the interventions and during the mixed meal tolerance test (MMTT). The panels to the left show profiles of hunger (A), prospective food consumption (C), nausea (E), and fullness (G). The gray area indicates time of intervention. MMTT was initiated at *t* = 105. The panels to the right (B, D, F, and H) show mean levels of the above‐mentioned variables during the MMTT. Data are mean ± SEM. Differences were analyzed by one‐way repeated‐measures (RM) ANOVA. Significant differences (*P* < 0.05) indicated by *(CON vs. IW) and tendencies for differences (*P* < 0.10) indicated by (*^≠^) (CON/CW vs. IW). CON, control; CW, continuous walking; IW, interval walking.

When evaluating specific time points, IW tended to reduce hunger compared to both CW and CON 2 h into the MMTT (*P* < 0.10 for both; Fig. 1A). Moreover, IW resulted in increased fullness compared to CON immediately before the MMTT (*P* < 0.05; Fig. 1G). No other between‐trial differences were seen for any satiety variables at any time point (*P* > 0.05 for all). When mean values during the MMTT were compared, no differences between trials were seen (*P* > 0.05 for all comparisons).

No differences between trials were seen for any satiety variable the day after the intervention day (*P* > 0.05 for all comparisons, data not shown).

### Appetite‐related hormones, lactate, and paracetamol (Fig. 2)

No baseline differences were seen in appetite‐related hormones, lactate, or paracetamol concentrations between trials.

**Figure 2:**
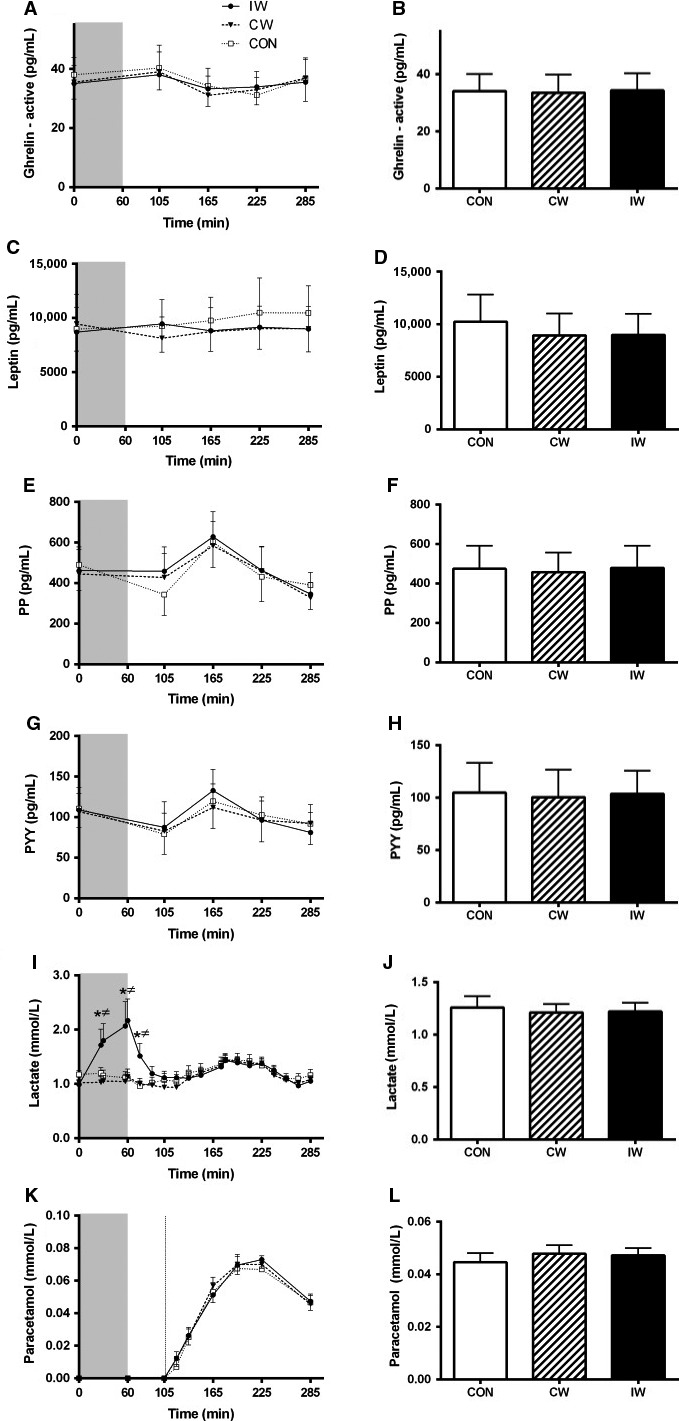
Appetite‐related hormones, blood lactate, and paracetamol measured before and after the interventions and during the mixed meal tolerance test (MMTT). The panels to the left show profiles of active ghrelin (A), leptin (C), PP (E), PYY (G), blood lactate (I), and paracetamol (K). The gray area indicates time of intervention. MMTT was initiated at *t* = 105. The panels to the right (B, D, F, H, J, and L) show mean levels of the above‐mentioned variables during the MMTT. Data are mean ± SEM. Differences were analyzed by one‐way repeated‐measures (RM) ANOVA. Significant differences (*P* < 0.05) indicated by *(CON vs. IW) and ^≠^(CW vs. IW). CON, control; CW, continuous walking; IW, interval walking; PP, pancreatic polypeptide; PYY, Peptide YY.

Blood lactate levels were increased during and 15 min after IW compared to both CW and CON (*P* < 0.05 for all comparisons; Fig. 2I), with no differences between CW and CON. During the MMTT, no differences between any trials were found (*P* > 0.05 for all comparisons).

No between‐trial differences were seen for any appetite‐related hormones or paracetamol, neither when evaluating specific time points, nor when mean values during the MMTT were compared (*P* > 0.05 for all comparisons).

### Ad libitum and free‐living energy intake (Fig. 3)

**Figure 3:**
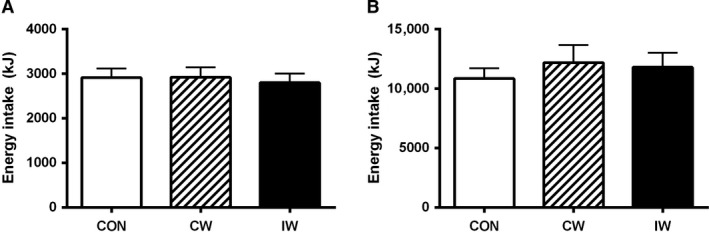
Energy intake measured during the ad libitum meal (A) and during the postintervention free‐living period (B). Data are mean ± SEM. Differences were analyzed by one‐way repeated‐measures (RM) ANOVA. No statistical significant differences between trials were found. CON, control; CW, continuous walking; IW, interval walking.

No significant between‐trial differences were seen in energy intake during the ad libitum meal (*P* > 0.05; Fig. 3A). Likewise, no between‐trial differences were seen in free‐living energy intake at the intervention day, the day after the intervention day or the combination of these two (*P* > 0.05; Fig. 3B).

Likewise, when subtracting energy expenditure during the interventions from total postintervention energy intake at the intervention day and the day after, no significant between‐trial differences were seen (CON = 13.2 ± 0.9 mJ; CW = 13.1 ± 1.7 mJ; IW = 12.3 ± 1.5 mJ; *P* > 0.05 for all comparisons).

## Discussion

The most important finding from this study is that exercise, independent of peak exercise intensity, neither affects ad libitum meal nor free‐living energy‐intake in subjects with T2D from ~4 to 36 h after the exercise session. Conversely, exercise with higher peak intensity (IW) increased fullness shortly after the exercise session compared to the CON, and exercise with higher peak intensity tended to reduce hunger compared to both the CON and exercise with lower peak intensity (CW) 2 h into a liquid MMTT. However, none of these findings remained significant when evaluating the mean effects during the MMTT, neither did they translate into effects on energy intake during the postexercise period.

The apparent lack of coherence between the effects of IW on satiety on one side and energy intake on the other side is noteworthy. In this context, it may be speculated that timing is important. As such, the higher fullness after IW compared to CON was seen shortly after cessation of the exercise bout, whereas no differences between trials were encountered from 1 h 45 min after completion of the interventions (Fig. 1G). Also, the tendency for a lower hunger sensation after IW compared to both CW and CON was observed 2 h into the MMTT, whereas no differences were seen immediately before initiation of the ad libitum meal (Fig. 1A). Since the effects of exercise on satiety is transient and dynamic (King et al. 2013; Williams et al. 2013; Hazell et al. 2016), it may be speculated that if the ad libitum meal had been served a shorter period after the intervention was performed, another outcome would have occurred. As such, bearing the increased fullness observed shortly after IW compared to CON in mind, a delayed and therefore potentially lower total energy‐intake after IW might have been seen in real‐life conditions. Moreover, exercise intensity may influence food preference (Klausen et al. 1999), and whereas the free‐living diet records did not indicate that this was the case in our study, we cannot rule out that the serving of a buffet instead of a fixed menu for the ad libitum meal would have changed the outcome. Finally, it must be acknowledged that the influence of exercise on energy intake and appetite regulation shows a substantial interindividual variability (King et al. 2012). Whereas a priori sample size calculations on ad‐libitum energy intake were performed and an acceptable power (0.82) based on these calculations was achieved in this study, it must be noted that this was based on a high correlation coefficient (0.91). In this study, the correlation coefficient between IW and CON was substantially lower (0.68), potentially indicating that intra‐individual differences in energy intake are affected by diabetes status. As such, we were most likely underpowered to detect any potential differences in energy‐intake between interventions.

The absolute energy‐intake (both in the ad libitum meal and during the free‐living period) was not reduced after the exercise sessions, but neither was it increased when compared to CON. With the increased energy consumption seen with the exercise sessions, this would theoretically induce negative energy balance in the exercise trials compared with CON. There was, however, also no significant differences in the relative energy intake (postexercise energy intake corrected for the energy cost of exercise above the resting level) (Pomerleau et al. 2004) between any of the trials, but given that free‐living, self‐reported diet records are prone to substantial uncertainties (Samuel‐Hodge et al. 2004), and given that huge differences in reported energy intake in the postintervention free‐living period (ranging from 5.7 to 24.8 mJ between subjects) were reported, the variance between subjects in this study was probably too large to detect any potential intervention‐induced differences. Thus, a statistical type 2 error might be considered. In this respect, the insignificant differences in relative energy intake should be highlighted with relative energy intake after IW being 0.9 ± 1.6 mJ and 0.8 ± 1.9 mJ lower than CON and CW, respectively.

Gastric emptying, which is tightly associated with hunger sensation (Janssen et al. 2011), has been found to be reduced after exercise and intensity has been suggested to be of major importance for this effect (Thivel et al. 2014; Horner et al. 2015). As such, this might potentially explain differences in satiety variables between CW and IW. However, no differences in gastric emptying during the MMTT were seen between any of the trials, indicating that this was not the case (Fig. 2K and L).

Our findings are somewhat conflicting with earlier studies. As such, Sim et al. found that interval‐type exercise with anaerobic peak intensity reduced both ad libitum and free‐living energy‐intake compared with both no exercise and continuous exercise with lower peak intensity. Moreover, they found that interval‐type exercise with aerobic peak intensity reduced ad libitum energy‐intake compared with no exercise. Despite the reason for the discrepancy seen between the study by Sim et al. and our study is not completely clear, several differences between the two studies should be highlighted. First, the study population (healthy vs. T2D subjects) is different and whereas exercise has been suggested to influence satiety and appetite‐related hormones in subjects with T2D (Knudsen et al. 2013; Heden et al. 2016), we and others have shown that appetite regulation is impaired and altered with hyperglycemia and T2D (Knudsen et al. 2013, 2014; Erdmann et al. 2005). Second, the peak exercise intensity was lower in our study compared to the study by Sim et al. (2014), and since this study reported that postexercise energy‐intake was successively reduced with increasing peak exercise intensity, the lacking effects of exercise on energy‐intake in our study may be due to too low peak intensity. Third, the timing of the ad libitum meal is different between the two studies (70 vs. 225 min after cessation of the exercise bout), and given the above‐mentioned dynamic and transient nature of exercise‐induced effects on satiety and appetite‐related hormones, this may also have influenced the differential results. However, since Sim et al. (2014) found that the energy‐intake was reduced for 2 days after the exercise session with highest peak intensity, this factor cannot completely explain the differential results. Lastly, it must be noted that other studies, carried out in nondiabetic subjects, have also found that exercise with higher peak intensity does not decrease (Holliday and Blannin 2017) and may even lead to higher energy‐intake than exercise with lower peak intensity (Pomerleau et al. 2004; Bilski et al. 2013).

In summary, this study has shown that exercise does not have a major influence on ad libitum and free‐living energy‐intake when evaluated ~4–36 h after the exercise bout in subjects with T2D. Conversely, some indications were found that interval‐type exercise with higher peak intensity increased fullness compared to no exercise shortly after the exercise bout, and that interval‐type exercise with higher peak intensity reduced hunger ~3 h after the exercise bout compared with both continuous‐type exercise with lower peak intensity and no exercise.

## Conflict of Interest

There is no conflict of interest that could be perceived as prejudicing the impartiality of the research reported.
